# An *in-silico* approach to studying a very rare neurodegenerative disease using a disease with higher prevalence with shared pathways and genes: Cerebral adrenoleukodystrophy and Alzheimer’s disease

**DOI:** 10.3389/fnmol.2022.996698

**Published:** 2022-09-27

**Authors:** Yu Jeong Shim, Min Kyoung Shin, Junghyun Jung, Bongseong Koo, Wonhee Jang

**Affiliations:** ^1^Department of Life Science, Dongguk University, Goyang-si, South Korea; ^2^BPgene, Seoul, South Korea

**Keywords:** adrenoleukodystrophy, Alzheimer’s disease, shared pathway, meta-analysis, neurodegenerative disease

## Abstract

Cerebral adrenoleukodystrophy (cALD) is a rare neurodegenerative disease characterized by inflammatory demyelination in the central nervous system. Another neurodegenerative disease with a high prevalence, Alzheimer’s disease (AD), shares many common features with cALD such as cognitive impairment and the alleviation of symptoms by erucic acid. We investigated cALD and AD in parallel to study the shared pathological pathways between a rare disease and a more common disease. The approach may expand the biological understandings and reveal novel therapeutic targets. Gene set enrichment analysis (GSEA) and weighted gene correlation network analysis (WGCNA) were conducted to identify both the resemblance in gene expression patterns and genes that are pathologically relevant in the two diseases. Within differentially expressed genes (DEGs), GSEA identified 266 common genes with similar up- or down-regulation patterns in cALD and AD. Among the interconnected genes in AD data, two gene sets containing 1,486 genes preserved in cALD data were selected by WGCNA that may significantly affect the development and progression of cALD. WGCNA results filtered by functional correlation via protein–protein interaction analysis overlapping with GSEA revealed four genes (*annexin A5*, *beta-2-microglobulin*, *CD44 molecule*, and *fibroblast growth factor 2*) that showed robust associations with the pathogeneses of cALD and AD, where they were highly involved in inflammation, apoptosis, and the mitogen-activated protein kinase pathway. This study provided an integrated strategy to provide new insights into a rare disease with scant publicly available data (cALD) using a more prevalent disorder with some pathological association (AD), which suggests novel druggable targets and drug candidates.

## Introduction

Neurodegenerative diseases (NDs) are a class of disorders that mainly affect the central nervous system (CNS) and are characterized by progressive loss of the structure and/or function of neurons (Nussbaum and Ellis, 2003). Recently, the number of patients suffering from NDs such as Alzheimer’s disease (AD), Huntington’s disease, Parkinson’s disease, and amyotrophic lateral sclerosis has been rapidly increasing worldwide (Hou et al., 2019). Significant efforts have been dedicated to developing medications that can cure NDs; however, no current therapeutics can completely cure these diseases. Even reversing damage is improbable, only a few treatments can slow the progression or alleviate these diseases’ symptoms (Crous-Bou et al., 2017). If the exact pathological pathways can be identified, it may help develop therapeutic agents that can cure NDs. The most frequent form of NDs is dementia, the most prevalent type of which is AD (Nussbaum and Ellis, 2003). In the United States about 5.8 million Americans of all ages reportedly have AD-type dementia, 200,000 of whom have AD under the age of 65 years (Alzheimer’s Association, 2019). The percentage of AD patients within the population is expected to increase by 6.7–30.8% depending on the state by 2025 compared to 2020 (Alzheimer’s Association, 2021).

X-linked adrenoleukodystrophy (ALD) is a rare ND characterized by fatal progressive cerebral demyelination and/or spinal cord neurodegeneration (Fourcade et al., 2008). The ALD phenotypes range from rapidly progressing childhood cerebral form to adrenomyeloneuropathy (AMN) with/without cerebral involvement in adults (Berger et al., 2014). ALD is caused by an abnormality in the *adenosine triphosphate binding cassette subfamily D member1* (*ABCD1*) gene (Xq28) that encodes an integral peroxisomal membrane protein (Fourcade et al., 2008). Childhood cerebral ALD (cALD), which develops in boys aged 5–12 years, accounts for 35% of all ALD patients (Berger et al., 2014). Symptoms of childhood cALD include autoimmune response, strong inflammatory demyelination, and rapid progression of neurological dysfunction, leading to death within a few years (Moser et al., 1992).

One feature of all ALD is the accumulation of very long-chain fatty acids (VLCFA; ≥ C22) caused by impaired peroxisome β oxidation (Moser et al., 1992; Berger et al., 2014). The accumulation of saturated VLCFA was also found in the cortex of AD patients (Kou et al., 2011). VLCFA aggregate throughout the body with the most severe accumulation in the white matter of the brain and adrenal glands, causing neurological problems and adrenal insufficiency (Fourcade et al., 2008; Berger et al., 2014). VLCFA was reportedly a potential risk factor contributing to neurodegeneration by inducing nerve cell damage through mitochondrial dysfunction (Schönfeld and Reiser, 2016; Nury et al., 2020). VLCFA levels can be lowered by oral administration of oleic acid (C18: 1) and erucic acid (C22: 1) at a 4:1 ratio, which is known as Lorenzo’s oil. Erucic acid is an important ligand of peroxisome proliferator-activated receptor δ, the activation of which directly inhibits neuronal cell death and alleviates neuro-inflammation in AD (Moser et al., 2007; Sassa et al., 2014; Altinoz et al., 2018; Altinoz and Ozpinar, 2019). Despite the link, very few studies have focused on the commonalities between ALD and AD.

Studies on rare diseases such as ALD generally have hardships of having a small sample size due to the low prevalence among the population, leading to difficulties in drug development (Engelen et al., 2014). Meta-studies merge datasets from individual studies to increase their sample size, thereby increasing statistical power, allowing the identification of novel pathways that cannot otherwise be found in separate studies. Comparing a rare disease with a more highly prevalent disease that shares a common pathway enables the designing of a novel drug that may act on both diseases (Goh et al., 2007).

As the development of novel *in silico* tools for analyzing genetic diseases arrives, studies are also actively being conducted to understand the biological meaning of a disease based on gene expression (Subramanian et al., 2005; Langfelder and Horvath, 2008). GSEA allows single-gene expression data to be compared with a distinct type of gene set conveying the biological roles and characteristics of other diseases. In addition to GSEA, a weighted gene co-expression network analysis (WGCNA) is a bioinformatic application for finding co-expression patterns between genes by constructing a network and is used to compare clustered genes with another set of genes. These are powerful analytical tools that can be used to investigate various diseases, even rare diseases, with which several studies have successfully elicited genetic markers (Jung et al., 2019; Bottero et al., 2021; Kim et al., 2021). After identifying these markers, drug-repositioning can be performed to develop novel drug candidates.

**FIGURE 1:**
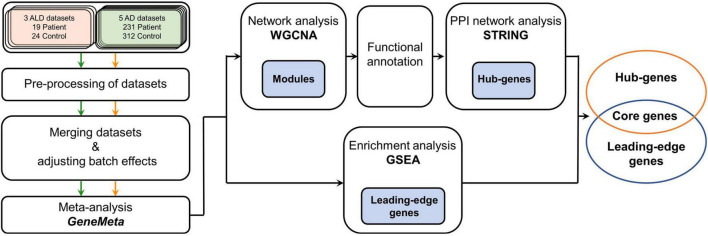
Workflow of the meta-analysis in this study. Green, orange, and black arrows indicate how cALD, AD, and the meta-analysis datasets were processed, respectively. The meta-analysis using GeneMeta was performed separately for two diseases, but the diseases were later analyzed together in the GSEA, WGCNA, and STRING processes. The blue painted boxes represent genes that were obtained from the corresponding analysis process, and those genes were used in the next analysis process. cALD, cerebral adrenoleukodystrophy; AD, Alzheimer’s disease; GSEA, gene set enrichment analysis; WGCNA, weighted gene co-expression network analysis; STRING, search tool for the retrieval of interacting genes/proteins; PPI, protein–protein interaction.

In this study, we designed a workflow to study cALD using AD data to discover meaningful pathogenetic pathways and novel genetic markers via combining two different bioinformatic approaches (Figure 1). GSEA was conducted to detect common differentially expressed genes (DEGs) to determine the similarities between the two diseases, followed by WGCNA to identify interconnected gene sets that have correlations with the pathogeneses of cALD and AD. The core genes were finally selected by overlapping genes from GSEA and WGCNA results, of which biological roles were revealed along with the pathogenic understanding of cALD. By integrating two analyses in a complementary manner, common marker genes and pathways in both diseases could be identified that can be suggested as putative targets for pathway-based drug repositioning.

## Materials and methods

### Data collection of cerebral adrenoleukodystrophy and Alzheimer’s disease

We searched for and downloaded microarray datasets of Homo sapiens from ArrayExpress.^1^ We used three datasets for cALD (E-MEXP-3288, E-GEOD-34309, E-GEOD-85804) and five datasets for AD (E-GEOD-36980, E-GEOD-5281, E-GEOD-29378, E-GEOD-48350, E-GEOD-34879) in this study that contained samples of postmortem brains and induced pluripotent stem cells (iPSCs) obtained from patients’ skin fibroblasts (cALD or AD) and from healthy control subjects (Liang et al., 2007; Israel et al., 2012; Schlüter et al., 2012; Wang et al., 2012; Berchtold et al., 2013; Miller et al., 2013; Hokama et al., 2014; Jang et al., 2016). The iPSC cells obtained from AD and control samples were induced into neurons, where only the induced neurons derived from AD patients showed a significant increase in three major biochemical markers of AD, amyloid-β, active glycogen synthase kinase-3β, and phosphorylated tau/total tau (Israel et al., 2012). The microarray datasets of iPSC samples were established from the fibroblasts cultured from skin biopsies of the patients and healthy controls. For the validation, an RNA sequencing (RNA-seq) data set (PRJNA422218) in which iPSC samples derived from somatic cells from childhood cALD patients were induced into brain microvascular endothelial cells was used (Lee et al., 2018).

### Data preprocessing

The downloaded raw datasets in Affymetrix platform (*.CEL files) and Illumina platform were normalized using the Robust Multi-array Average (RMA) algorithm and the neqc function in R package oligo and limma, respectively. The duplicate genes in the datasets were processed using the probe’s Entrez ID in the annotation package following Jung et al. (2017). The mean values were used for the data with identical Entrez IDs (Jung et al., 2018).

### Adjusting batch effects and computing *Z*-scores in merged data

The datasets were merged and adjusted the batch effect and by the R surrogate variable analysis (sva) package to maintain meaningful biological effects while eliminating non-biological effects that result from combining independent studies conducted in different environments (Johnson et al., 2006; Leek et al., 2012). A meta-analysis was conducted using the random effects model in the GeneMeta R package to obtain false discovery rates (FDRs) and *Z*-scores that represent the gene expression profiles in each disease (Choi et al., 2003). The *Z*-score was calculated to indicate how the expression of a single gene in a patient group is different from that of a control group in this study. That is, genes with positive values of the *Z*-score are expressed higher in the patient group compared with the control group, whereas genes with negative values are less expressed in the patient group compared with the control group.

### Gene set enrichment analysis

A fast GSEA R package was implemented for GSEA (Subramanian et al., 2005; Sergushichev, 2016). The *Z*-scores of the AD datasets were used to make a list of ranked genes. The DEGs of cALD were used as a set of genes to be analyzed and enrichment scores were calculated based on the ranked list. GSEA is designed to test multiple hypotheses for the similarity between ranked gene lists and a set of genes and has an algorithm to calculate enrichment scores by weighting the extreme (top or bottom) of the entire ranked list (Subramanian et al., 2005). The core members of the gene set with a high enrichment score were selected as leading-edge genes (Subramanian et al., 2005). For the functional annotation of modules, the hallmark genes from molecular signatures database (MsigDB^2^) were used as annotated gene sets (Liberzon et al., 2015).

### Weighted gene co-expression network analysis

Originally, a signed WGCNA is designed to cluster gene sets that solely consist of positively correlated genes based on Pearson correlation coefficients (Langfelder and Horvath, 2008). First, we performed a signed WGCNA using the merged datasets of AD (Jung et al., 2019; Kim et al., 2021). To describe this in detail, outliers of samples were eliminated by the hierarchical cluster method and the soft thresholding power (β) was calculated via scale-free topology analysis to a value of 10. Next, the adjacency matrix was converted into a topology overlap matrix to reflect the topology information on network formation. The modules were identified by the hierarchical cluster method and module eigengenes were calculated as summarized gene expression patterns of their respective modules. The modules were clustered with a minimum size of 30 genes. All modules were compared pair-wise, and pairs of modules showing high module eigengene correlations (r > 0.80) were merged. In order to find modules that showed correlations with cALD, module preservation analysis was conducted among the modules constructed from the AD dataset as the reference set and the merged cALD dataset as the test set (Langfelder et al., 2011). The evidence that a module is preserved is summarized by the *Z*-summary score, which was created by averaging the various preservation statistics of module robustness and reproducibility (Langfelder et al., 2011). All the above analyses were conducted in R (version 4.1.2).

### Protein–protein interaction network analysis

The search tool for the retrieval of interacting genes/proteins (STRING^3^.) was applied to investigate the connections between genes at the protein level (Szklarczyk et al., 2019). The STRING provides predictions of protein–protein interactions (PPIs) by taking a list of proteins as input, calculating confidence scores based on various evidence of interactions among proteins, and assigning uniform confidence scores to the same data set (Szklarczyk et al., 2019). The confidence level of the edge was adjusted to 0.7 and the nodes that were connected to fewer than two other nodes were deleted (Apostolakou et al., 2021). A PPI network constructed by STRING was visualized by cytoscape (version 3.9; Shannon et al., 2003). In the network, highly interconnected gene clusters were found by molecular complex detection (MCODE) with the cytoscape plugin (Bader and Hogue, 2003).

## Results

### Data collection of cerebral adrenoleukodystrophy and Alzheimer’s disease and identifying differentially expressed genes in cerebral adrenoleukodystrophy by meta-analysis

To conduct a meta-analysis that utilizes multiple datasets together, gene expression datasets were collected from EBI-arrayexpress that contained samples from healthy controls and patients (cALD or AD) who had not received any drug intervention. Only one RNA-seq dataset from cALD patients and three microarray datasets from cALD patients and control subjects were publicly available. One microarray dataset was obtained from the postmortem brains of control subjects and cALD patients. This dataset also included cerebral AMN (cAMN) patients since cAMN is a subtype that shows mild cerebral-specific symptoms of cALD. Two additional datasets were obtained from the samples of early passage cultures of iPSCs derived from the skin fibroblasts of control subjects and cALD patients. In total, 43 datasets were used for cALD (19 cALD and 24 control; Table 1). The AD microarray datasets were searched for in the same way as the cALD microarray datasets and five AD microarray datasets were selected. Four datasets were generated with postmortem brains and the other dataset produced by iPSCs-induced neurons. These neurons exhibited significantly higher levels of Aβ and phosphorylated tau, and thus mimicked a live AD patient’s brain (Israel et al., 2012). In total, 534 data were obtained from control subjects and AD patients who did not receive treatment (231 AD and 313 control; Table 2).

**TABLE 1:** cALD dataset.

ArrayExpress ID	Source	Platform	Number of samples		
			*cALD	Control	Total
E-MEXP-3288	Postmortem brain	Affymetrix GeneChip Human Genome HG-U133A	11	13	24
E-GEOD-85804	*iPSCs	Illumina HumanHT-12 V4.0 expression beadchip	3	3	6
E-GEOD-34309	*iPSCs	Affymetrix Human Genome U133A 2.0 Array	5	8	13

			19	24	43


*iPSC, induced pluripotent stem cells; cALD, cerebral adrenoleukodystrophy.

**TABLE 2:** AD dataset.

ArrayExpress ID	Source	Platform	Number of samples		
			*AD	Control	Total
E-GEOD-36980	Postmortem brain	Affymetrix Human Gene 1.0 ST Array	32	47	79
E-GEOD-5281	Postmortem brain	Affymetrix Human Genome U133 Plus 2.0 Array	87	74	161
E-GEOD-29378	Postmortem brain	Illumina HumanHT-12 V3.0 expression beadchip	17	16	33
E-GEOD-48350	Postmortem brain	Affymetrix Human Genome U133 Plus 2.0 Array	80	173	253
E-GEOD-34879	*iPSC-induced neurons	Illumina HumanHT-12 V4.0 expression beadchip	15	2	17

			231	312	543


*iPSC, induced pluripotent stem cells; AD, Alzheimer’s disease.

**FIGURE 2:**
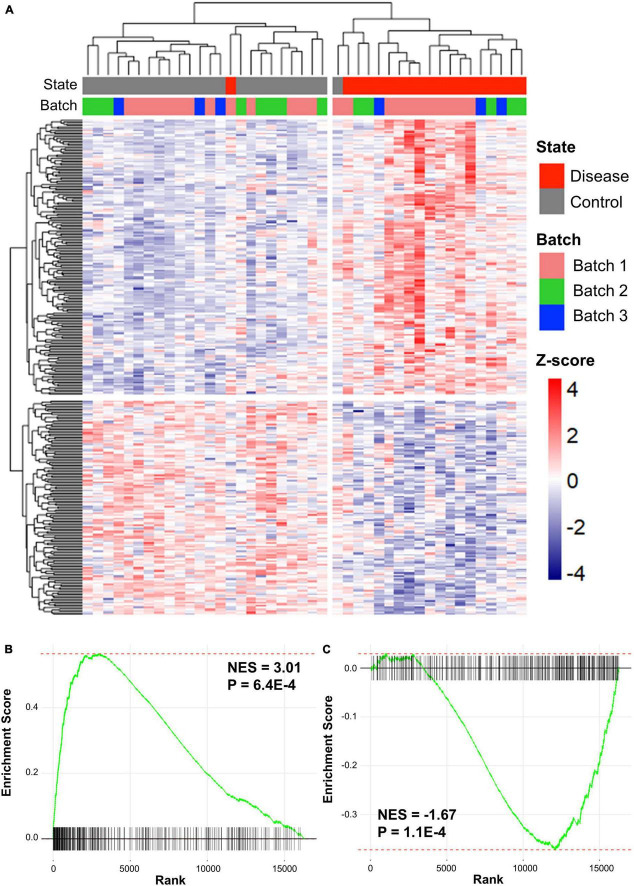
Clustered results of DEGs and GSEA plots of cALD and AD. **(A)** Heatmap showing expression patterns of DEGs in cALD (FDR < 0.05). The disease states of individuals are shown as red and gray bars, and the batch represents the three datasets of cALD as green, blue, and pink bars above the heatmap. The color inside the heatmap shows the *Z*-score of cALD. GSEA plot comparing the expression of the AD dataset to DEGs that were significantly up-regulated **(B)** and down-regulated **(C)** in cALD. The normalized enrichment score (NES) indicates the degree to which DEGs of cALD were overexpressed at the top or bottom of ranked expression in the AD dataset.

Preprocessing was performed for each dataset and the datasets were merged based on the disease type. Batch effects, which are non-biological variants caused by two merged datasets, were removed using Combat function in the sva R package. The *Z*-scores of genes in the two merged datasets were calculated using GeneMeta R package (Johnson et al., 2006). In total, 636 cALD DEGs were screened by the cALD *Z*-scores and FDRs of the genes for GSEA (|*Z*-score| > 0 and FDR < 0.05; Figure 2A). Among cALD DEGs, 317 and 319 genes were up- and down-regulated, respectively.

### Comparison of cerebral adrenoleukodystrophy and Alzheimer’s disease patients’ gene expression patterns

To determine whether cALD and AD have similar gene expression patterns in the brain, GSEA were conducted to examine two gene expression data sets from cALD and AD. GSEA is a gene rank-based analysis method extensively used in meta-analysis when evaluating two gene expression datasets. Therefore, GSEA was utilized to observe whether the expression of the cALD dataset was similar to that of the AD dataset (Subramanian et al., 2005). The *Z*-scores of the entire AD dataset (16,265 genes) were ranked from highest to lowest and then up- and down-regulated DEGs in cALD were examined to place them in the ordered gene list of AD. Using GSEA, the normalized enrichment scores (NES) of DEGs in cALD were calculated from the *Z*-scores of the same DEGs obtained from the AD gene list. The NES value increases when the DEG in cALD is ranked high in AD data and *vice versa*. The GSEA results showed that both up- and down-regulated DEGs were positively and negatively enriched with *p*-values < 0.05 (up-regulated DEGs: NES = 3.01 and *p*-value = 6.4 × 10^−4^ and down-regulated DEGs: NES = −1.67 and *p*-value = 1.1 × 10^−4^; Figures 2B,C (Subramanian et al., 2005). The leading-edge genes are key genes that contribute to the NES, which are considered shared DEGs in two diseases (Subramanian et al., 2005). We identified 144 and 122 up- and down-leading-edge genes in cALD and AD via GSEA (Supplementary Table 1). The GSEA results indicate that cALD and AD have similar gene expression patterns with significant NES and leading-edge genes.

### Identification of co-expressed gene modules in cerebral adrenoleukodystrophy and Alzheimer’s disease by weighted gene correlation network analysis

**FIGURE 3:**
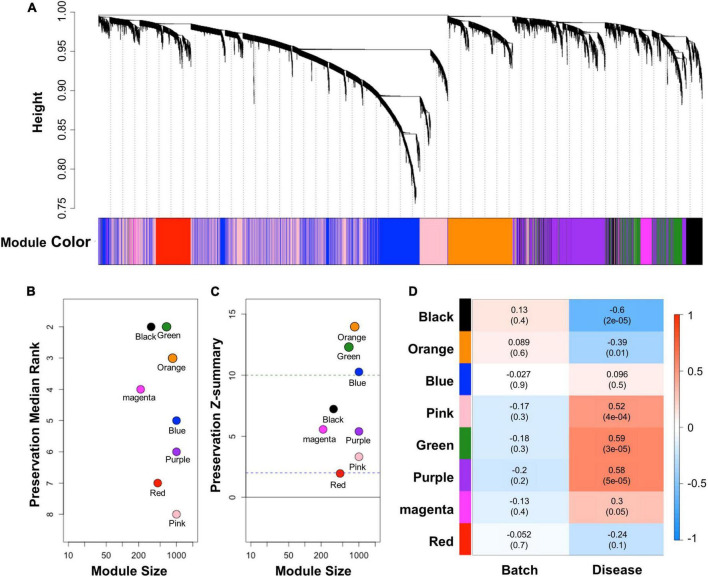
Results of WGCNA and module preservation analysis. **(A)** Dendrogram showing the modules obtained from the signed network based on the dissimilarity of the topology overlap matrix of the AD modules. A total of eight modules were clustered and are represented by color. The modules were sorted by their respective module size and aligned by preservation median rank **(B)** and preservation *Z*-summary score **(C)** against cALD data. The dashed line at 10 indicates a strong preservation threshold, whereas the dashed line at 2 indicates no preservation threshold. **(D)** Matrix showing the correlation of characteristics of the samples in the merged cALD dataset and the genes in the modules. The numbers in parentheses are their respective *p*-values.

WGCNA was conducted to comprehend the gene expression profile that is applicable in cALD from interconnected genes in AD. WGCNA provides network topology information and modules that indicate correlated gene sets by performing correlation network analysis on a high-dimensional dataset. We constructed a correlation weighted network of the AD dataset with preserved sign information of gene expression. Eight modules (black, blue, green, magenta, orange, pink, purple, and red) were detected by constructing a network of AD (Figure 3A). To identify applicable modules in cALD, we performed module preservation analysis using AD modules and the cALD dataset. Preservation median rank and *Z*-summary scores were obtained from preservation analysis and the scores were considered to have strong, weak-to-moderate, or little-or-no preservation when the score was > 10, 2–10, or < 2, respectively (Langfelder et al., 2011). The green and orange modules were highly preserved in the cALD dataset (green: 12 and orange: 14) and the other modules were moderately preserved in the cALD dataset (blue: 10; black: 7.2; magenta: 5.6; purple: 5.4; gold: 5.1; pink: 3.3; red: 2.0; Figures 3B,C). The Pearson correlation coefficients of both disease states and batches in the cALD dataset were calculated with the module eigengenes, which contains the expression profile of each module (Figure 3D). The *p*-value of the correlation coefficient indicates whether the correlation coefficient is significantly different from 0. The green and orange modules were considered significantly different in gene expression between the control and patient groups (green: r = 0.59 and *P* = 3 × 10^−5^; orange: r = −0.39 and *P* = 0.01) and the number of genes in the two modules were 652 and 834, respectively. The results of WGCNA indicated that the green and orange modules were important for the causal genetic relationship between cALD and AD.

### Preservation module related to immune process and cell death

**FIGURE 4:**
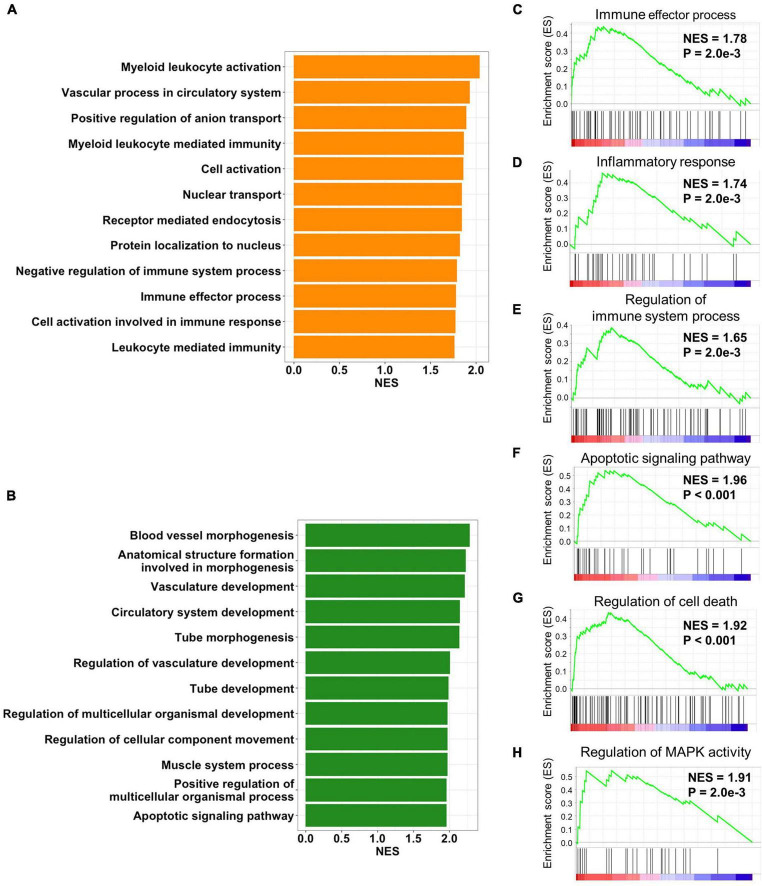
Biological processes of preserved modules. Bar plots represent *p*-values of biological process of gene ontology from **(A)** orange and **(B)** green modules. The top 12 biological processes with the most overlapped genes between the annotated gene list and module genes are displayed. The orange module was positively enriched with **(C)** the immune effector process, **(D)** inflammatory response, and **(E)** regulation of the immune system process. The green module was positively enriched with **(F)** the regulation of MAPK activity, **(G)** the regulation of cell death, and **(H)** the apoptotic signaling pathway.

In order to gain insights into the biological processes of the cALD-related preserved modules, functional annotation was performed using GSEA with gene ontology biological process gene sets. We performed gene annotation with biological process of gene ontology using green and orange modules and screened biological processes at *P* < 0.01. There were 24 and 53 biological processes that met the criteria of *P* < 0.01 in gene annotation of the green and orange modules, respectively. Biological processes were ordered by NES of overlapped genes between annotated modules and the genes constituting individual biological processes. In the orange module, the top 12 enriched biological processes were myeloid leukocyte activation (NES = 2.03 and *P* < 0.001), vascular process in circulatory system (NES = 1.92 and *P* = 2.0 × 10^−3^), positive regulation of anion transport (NES = 1.89 and *P* < 0.001), myeloid leukocyte-mediated immunity (NES = 1.86 and *P* < 0.001), cell activation (NES = 1.86 and *P* < 0.001), nuclear transport (NES = 1.84 and *P* < 0.001), receptor-mediated endocytosis (NES = 1.84 and *P* = 4.3 × 10^−3^), protein localization to nucleus (NES = 1.82 and *P* = 6.8 × 10^−3^), negative regulation of immune system process (NES = 1.78 and *P* = 6.8 × 10^−3^), immune effector process (NES = 1.78 and *P* = 2.3 × 10^−3^), cell activation involved in immune response (NES = 1.77 and *P* < 0.001), and leukocyte-mediated immunity (NES = 1.76 and *P* < 0.001; Figure 4A). In the green module, the top 12 enriched biological processes were blood vessel morphogenesis (NES = 2.28 and *P* < 0.001), anatomical structure formation involved in morphogenesis (NES = 2.22 and *P* < 0.001), vasculature development (NES = 2.21 and *P* < 0.001), circulatory system development (NES = 2.14 and *P* < 0.001), tube morphogenesis (NES = 2.13 and *P* < 0.001), regulation of vasculature development (NES = 2.00 and *P* < 0.001), tube development (NES = 1.98 and *P* < 0.001), regulation of multicellular organismal development (NES = 1.97 and *P* < 0.001), regulation of cellular component movement (NES = 1.97 and *P* < 0.001), muscle system process (NES = 1.97 and *P* < 0.001), positive regulation of multicellular organismal process (NES = 1.96 and *P* < 0.001), and apoptotic signaling pathway (NES = 1.97 and *P* < 0.001; Figure 4B).

As a result of functional annotation of the orange module, immune effector process, inflammatory response (NES = 1.74 and *P* = 2.0 × 10^−3^), and the regulation of immune system process (NES = 1.65 and *P* = 2.0 × 10^−3^) were immune-related pathways related to the pathogenesis of both diseases (Figures 4C–E). The green module was enriched with cell death-related processes including the apoptotic signaling pathway and the regulation of cell death (NES = 1.92 and *P* < 0.001). In addition, mitogen-activated protein kinase (MAPK) activity (NES = 1.91 and *P* = 2.0 × 10^−3^), which was involved in inflammation and cell death, was also enriched in the green module (Figures 4F–H). Although the role of the MAPK pathway in cALD has not been thoroughly revealed, it is known to regulate several cellular processes including development, apoptosis, and inflammation in AD (Cui et al., 2013; Thei et al., 2018).

### Identification hub-genes of cerebral adrenoleukodystrophy and Alzheimer’s disease by protein–protein interactionnetwork analysis

**FIGURE 5:**
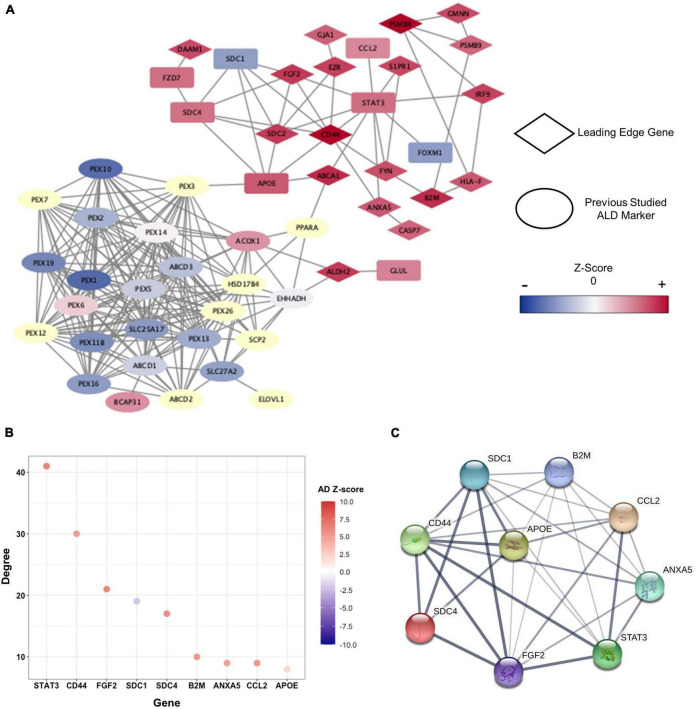
Clusters within the PPI network and characteristics of hub-genes. **(A)** PPI network presented by cALD-related genes and genes of |*Z*-score| > 2 in cALD and AD among green and orange modules. The leading-edge genes are indicated by rhombi, and previously studied cALD marker genes are indicated by ellipses. The range of *Z*-score values in the cALD dataset of the nodes is represented by a color bar at the right. The undetected genes in the microarray are shown as pale-yellow nodes and were not used in meta-analysis. **(B)** The degree of hub-genes was obtained using green and orange modules. The degree of each node represents the total number of connections to other nodes in a network, and the colors of dots represent *Z*-scores of AD. **(C)** Protein interaction among hub-genes clustered around APOE. The thickness of the connected lines represents the strength of correlation between two hub-genes.

To identify hub-genes that affect the pathogenesis of cALD and AD, we conducted PPI network analysis by STRING to discover the connection of genes at the protein level. STRING provides interaction information between gene-encoded proteins using a network that contains proteins’ structural and functional information. The numbers of genes in the green and orange modules were reduced to 167 based on | *Z*-score| > 2 in cALD and AD (Bai et al., 2020). In addition, we obtained 38 genes that correspond to relevance score > 2 in cALD from the GeneCards^4^ to identify how the selected genes interact with the known cALD genes, of which 19 genes overlapped (Supplementary Table 2; Rappaport et al., 2017). As described above, PPI network analysis was conducted using 186 genes composed of module genes and known cALD genes (Figure 4A). The entire PPI network was formed by 106 genes excluding unlinked genes, and the *p*-value was calculated as 2.22 × 10^−16^. The *p*-value suggested that the analyzed genes have statistically higher interactions and shared more biologically significant links than a random geneset of the same size and linkage distribution (Figure 5A). *apolipoprotein E* (*APOE*; cALD *Z*-score = 2.97 and AD *Z*-score = 2.07) and *ATP binding cassette subfamily A member 1* (*ABCA1*) genes served as bridges between cALD-related genes and the module genes. *ABCA1* (cALD *Z*-score = 3.86 and AD *Z*-score = 5.05) was a leading-edge gene in this study.

We then utilized MCODE to detect highly interconnected genes—called hub-genes—as clusters based on the topology of the constructed PPI network (Supplementary Table 3). *APOE* was identified as a seed node around which the remaining eight genes, namely *annexin A5* (*ANXA5*), *beta-2-microglobulin* (*B2M*), *C-C motif chemokine ligand 2* (*CCL2*), *CD44 molecule* (*CD44*), *fibroblast growth factor 2* (*FGF2*), *syndecan 1* (*SDC1*), *syndecan 4* (*SDC4*), and *signal transducer and activator of transcription 3* (*STAT3*) were clustered. *APOE* is a well-known major genetic risk factor for late-onset AD. Even though the correlation between *APOE* and cALD pathogenesis has not been completely revealed, a recent proteomic study on the cerebrospinal fluid of active cALD patients showed that APOE is an inflammatory marker for the disease (Orchard et al., 2019). The hub-genes were highly interconnected among themselves and were also connected with other gene networks of the green and orange modules (Figures 5B,C). The distinction between control subjects and cALD patients in RNA-seq data were validated from the expression of hub-genes via principal component analysis (PCA; Supplementary Figure 1). This confirms that the hub-genes of cALD and AD were reliably discovered in this study.

To identify genes that have robust associations with the pathogenesis of diseases, four core genes—*B2M*, *CD44*, *FGF2*, and *ANXA5*—were selected from the hub-genes that overlapped with the GSEA results. Among the biological processes we discovered (Figures 4C–H), core genes were closely related to inflammation and apoptosis. *B2M* (cALD *Z*-score = 3.81 and AD *Z*-score = 5.35) serves as an inflammatory marker in the CNS (Liu et al., 2014; Topçiu-Shufta et al., 2016), and *CD44* (cALD *Z*-score = 4.67 and AD *Z*-score = 4.96) is positively correlated with apoptosis and inflammation by regulating cytokine expression (McKallip et al., 2002). *FGF2* (cALD *Z*-score = 3.58 and AD *Z*-score = 6.10) increased susceptibility to oxidative stress that induces neuronal cell death in astrocytes. The level of *ANXA5* (cALD *Z*-score = 2.98 and AD *Z*-score = 4.79), which is associated with familial late-onset AD from whole exome sequencing, in the cerebrospinal fluid of AD patients increased proportionally with the severity of the disease state as Aβ accumulates, playing a protective role against Aβ toxicity (Zhang et al., 2019; Bartolome et al., 2020).

## Discussion

*In-silico* approaches can be applied as tools to expand our understanding of diseases and suggest new therapeutic targets, which may reduce the time spent on laborious and time-consuming pre-screening processes. Meta-analyses combine multiple studies to increase both the sample size and statistical power and can particularly be effective when studying rare diseases as they usually consist of studies with small samples (Bradburn et al., 2007). cALD is a rare disease characterized by complex metabolic disorders in the cerebral and adrenal cortexes, the exact pathogenesis and molecular mechanisms of which remain unclear due to the near absence of data. Currently available treatment options include, but are not limited to, medication for relieving stiffness and seizures, Lorenzo’s oil (Moser et al., 1992), and stem cell transplantation (Cartier et al., 2009). In order to develop novel therapeutic options, a deeper understanding of the disease is required by overcoming the problem of data insufficiency (Berger et al., 2014). In comparison, numerous studies have been conducted because of the worldwide prevalence of AD, resulting in the discovery of various novel pathogenic mechanisms and treatments. Based on the fact that both diseases are NDs and share a common remedy (erucic acid), the purpose of this study was to utilize the large amount of data on AD to discover new pathological targets for cALD. Therefore, we analyzed the expression profiles and co-expression network of cALD and AD in parallel based on meta-analyses and revealed that the two diseases share distinct gene expressions, leading to the discovery of novel genes that may affect the pathogenesis of cALD (Figure 1).

To conduct our meta-analysis, we first constructed individual datasets for cALD and AD. As far as we know, other than one RNA-seq data obtained from iPSC-induced endothelial cells from cALD patients that we used for validation, only one microarray data composed of postmortem brain samples from cALD was available in the public database (Table 1). To increase the quantity of data and to estimate the disease state of the brain closer to its living state (Manchia et al., 2017), we used gene expression profiles of iPSCs from cALD patients. Wang et al. reported the suggestible transcriptome-level coherence between iPSCs of cALD patients and the known pathogenetic characteristics of cALD, including neuro-inflammation and peroxisome abundance (2012). One pathogenetic hypothesis of cALD includes that mutation in *ABCD1* gene impacts the endothelia of the brain microvasculature, leading to inflammatory demyelination in the brain (Lauer et al., 2017). Gene mutations promote the accumulation of VLCFA, oxidative stress, and cell death (Wiesinger et al., 2013). Since this study was mainly focused on the neuronal pathophysiology of cALD, the RNA-seq dataset of iPSC-induced endothelial cells was excluded from the meta-analysis and only used as a validation dataset. In addition, the cALD-related genes provided by the GeneCards were used to validate the results of WGCNA conducted in this study by utilizing the results of previous studies. Among cALD-related genes, genes without *Z*-scores for cALD and AD were not detected or designed to be undetected in at least one cALD microarray dataset (Supplementary Table 2). Even after adding these data, the insufficiencies remained in the cALD data.

There are abundant RNA-seq and microarray data on AD brains; however, there are almost no publicly available data obtained from live brains as far as we know. Most data were generated using postmortem brains due to the special nature of the brain itself. The iPSC-induced neuron dataset from AD patients showed conforming biological characteristics with the original neurons of AD patients (Table 2; Almeida et al., 2013; Chen et al., 2013). Therefore, the aforementioned dataset was included when constructing a combined gene expression matrix for AD in this study. There was much less RNA-seq AD data than that of the microarray dataset, consisting of 71 patients and 87 controls, which is less than one-third of the microarray dataset in this study (Supplementary Table 4). The merged data including an AD RNA-seq dataset had even fewer genes than the AD microarray dataset. In order to minimize the loss of genes that may provide potential signatures, merged data were constructed with only microarray platforms.

After data preparation, two statistically powerful approaches (GSEA and WGCNA) based on meta-analysis were performed to investigate the sharing transcriptomic aspects of cALD and AD. Gene set-level correlation analysis between cALD and AD was performed using GSEA, which suggested 144 up- and 122 down-regulated leading-edge genes that can be regarded as key driver genes in the shared genetic mechanisms (Figure 2). To simultaneously consider the co-expression structure of cALD and AD, WGCNA was conducted by constructing a gene network (Figure 3A). Among the eight modules identified in AD data, the network connectivity and correlation structures of the green and orange modules were conserved and significantly correlated with the disease state of cALD (*Z*-summary score > 10 and *P* < 0.05), which suggests a shared co-expression structure between cALD and AD (Figure 3). Among the significantly enriched biological pathways from both cALD and AD, immune response and cell death are known to occur via oxidative stress associated with the MAPK signaling pathway in AD (Figure 4; Kamat et al., 2014). Most biological processes of functional annotation were related to the pathogenesis of cALD and AD; the results suggested that most functional annotation results were in line with previous studies. The accumulation of VLCFA is reported to be the key contributor to oxidative stress in cALD, where excessive oxidative stress causes neuro-inflammation and eventually leads to the apoptosis of neuronal cells in cALD and AD (Behl, 1999; Berger et al., 2014). Even though the role of MAPK signaling pathway was not clearly revealed in cALD, the MAPK pathway may be suggested as an intermediating mechanism between oxidative stress and immune response and/or cell death considering the pathway-level similarity of cALD and AD.

Through systemic analysis of the PPI network, nine hub-genes were identified, led by *APOE* as the seed node of the cluster (Figure 5). APOE is a lipid transport protein that regulates the lipid metabolism, oxidative stress, neurite outgrowth, and the mitochondrial metabolism (Orchard et al., 2019). As the association between *APOE* and AD has been well-established for decades, the APOE’s role as a potential biomarker for cALD has been recently proposed (Orchard et al., 2019). Considering the statistical significance from GSEA and WGCNA along with network topology from the PPI network analysis, *B2M*, *CD44*, *FGF2*, and *ANXA5* were identified as core genes. In accordance with the result from the functional enrichment analysis of WGCNA modules, the core genes were related to inflammation, apoptosis, and MAPK in AD. In detail, B2M plays a critical role in inflammation and apoptosis and has been demonstrated to induce cognitive impairment in AD (Topçiu-Shufta et al., 2016; Zhong et al., 2020). *CD44* encodes cell-surface glycoproteins involved in cell–cell and cell–extracellular matrix interaction, where its expression on immune cells is known to regulate inflammation and apoptosis in CNS (McKallip et al., 2002). By contributing to a variety of biological activities, FGF2 plays significant role in apoptosis and differentiation in CNS and can activate the MAPK pathway (Liu et al., 2014). While four core genes were reported as the causal genetic risk factors for AD, the expression level of *ANXA5* is known to have a negative correlation with AD risk. *ANXA5* encodes protein that has inhibitory effects on inflammation and early apoptosis, contributing to tissue homeostasis (Bartolome et al., 2020). Despite the insufficient evidence explaining the pathological roles of these four core genes in cALD, the results of this study showed the potential of the core genes as the biomarker candidates for cALD. We finally suggested the four genes as novel targets for cALD as they are closely related to the common pathological phenotypes of cALD and AD.

In conclusion, the knowledge on pathological mechanisms and genes of cALD was successfully expanded through combination of the results of GSEA and WGCNA using AD datasets based on meta-analysis. The study showed the shared pathway between cALD and AD, finally determining the novel target genes of cALD. These findings can help fill in gaps in previously unknown pathways in cALD, which were found through two main approaches: increasing sample sizes for cALD and comparing the gene expression patterns of cALD with a disease with a higher prevalence and some common features, AD. The putative gene markers can potentially be applied not only to therapeutic targets or genetic diagnosis, but also to the potential drug repositioning for cALD, which is further augmented by rapid study on AD (Ashburn and Thor, 2004). To the best of our knowledge, this is the first meta-analysis to discover genetic similarities and common pathological factors derived from the correlation between cALD and AD. Because this study was conducted using *in silico* analyses, rigorous validation through functional studies might be necessary. Despite this limitation, this research demonstrated an approach for studying pathologically relevant diseases by deriving novel biological meaning of a very rare disease, which suggests a potential extension for various approaches.

## Data availability statement

The datasets presented in this study are included in the article/Supplementary material, further inquiries can be directed to the corresponding author.

## Author contributions

YS devised this study and conducted *in silico* analysis. YS and MKS wrote the manuscript. JJ, BK, and WJ supervised the research. WJ edited and gave final approval of the manuscript. All authors contributed to the article and approved the submitted version.

## Footnotes

1
https://www.ebi.ac.uk/arrayexpress/

2
http://software.broadinstitute.org/gsea/msigdb

3 https://string-db.org/, version 11.5

4
www.genecards.org/
