# Supplementary material for: An in-silico approach to studying a very rare neurodegenerative disease using a disease with higher prevalence with shared pathways and genes: Cerebral adrenoleukodystrophy and Alzheimer’s disease
Source: Front Mol Neurosci. 2022 Sep 27;15:996698. doi: 10.3389/fnmol.2022.996698 (PMC9553843; doi:10.3389/fnmol.2022.996698)
Supplement: Supplementary file 1 [file Data_Sheet_1.docx]

Supplementary Material

# Supplementary Figures


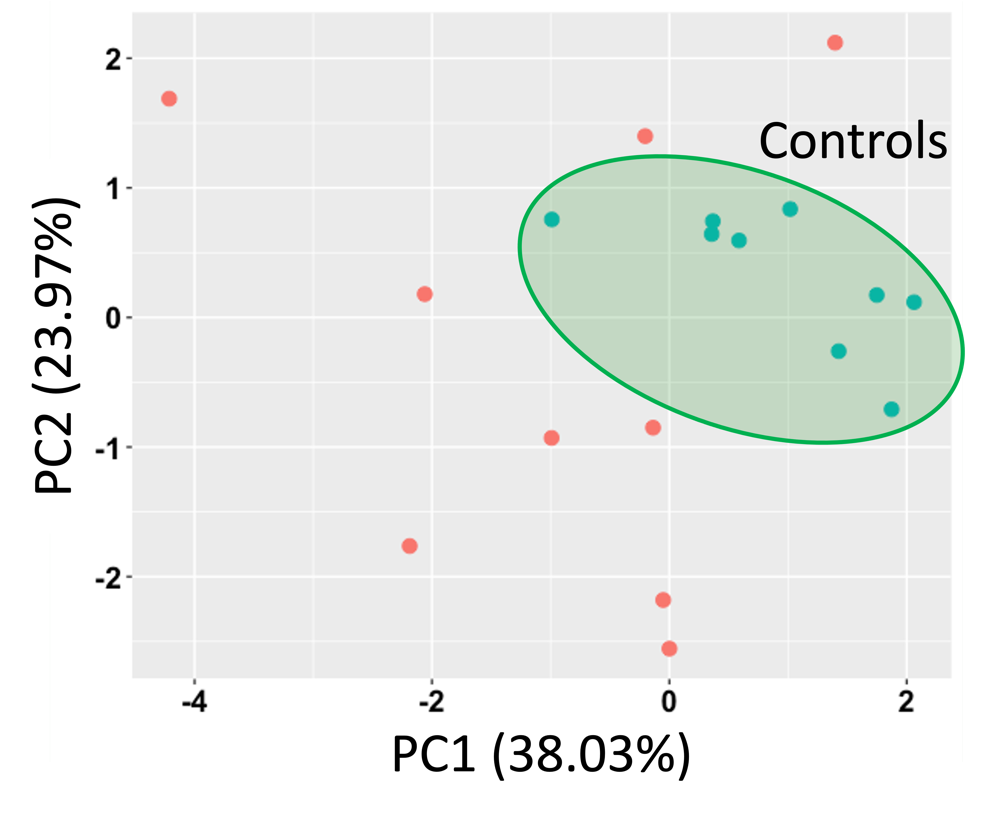


### Supplementary Figure 1. Scatter plot of PCA showing a distinct separation in the expression levels of hub-genes between patients with cALD and healthy controls from RNA-seq data of cALD (PRJNA422218).

### PCA plot with first principal component (PC1) and second principal component (PC2) shows summarized information of gene expressions in the cALD RNA-seq data with dimension reduction. X-axis represents PC1, which represents the direction of maximum variation through the data. Y-axis shows PC2, which is orthogonal to the PC1 and presents the second highest variation of the data. The scores of the PC1 and PC2 were standardized with a mean of 0 and scaled to the unit variance.

## Supplementary Tables

# Supplementary Table 1. Gene expression profiles of leading-edge genes

| Symbol | Entrez ID | Z-scores | | Leading-edge Gene |
| --- | --- | --- | --- | --- |
|  |  | **ALD** | **AD** |  |
| ABCA1 | 19 | 3.868 | 5.058 | Up |
| ABHD4 | 63874 | 3.113 | 5.247 | Up |
| ADGRG1 | 9289 | 4.289 | 4.995 | Up |
| AEBP1 | 165 | 2.947 | 8.227 | Up |
| AHNAK | 79026 | 3.097 | 9.154 | Up |
| ALDH2 | 217 | 3.541 | 2.947 | Up |
| ANXA1 | 301 | 4.245 | 4.286 | Up |
| ANXA2 | 302 | 3.652 | 5.681 | Up |
| ANXA5 | 308 | 2.981 | 4.79 | Up |
| ARPC1B | 10095 | 3.423 | 5.042 | Up |
| ATP1B2 | 482 | 2.87 | 4.09 | Up |
| ATP6V0E1 | 8992 | 3.892 | 5.391 | Up |
| AXL | 558 | 3.477 | 4.854 | Up |
| B2M | 567 | 3.802 | 5.354 | Up |
| BACH1 | 571 | 3.557 | 5.344 | Up |
| BAG3 | 9531 | 3.144 | 6.221 | Up |
| BMPR1A | 657 | 3.046 | 7.742 | Up |
| BST2 | 684 | 2.928 | 4.674 | Up |
| BTG1 | 694 | 3.649 | 4.719 | Up |
| C1S | 716 | 3.089 | 3.452 | Up |
| CASP7 | 840 | 2.753 | 5.437 | Up |
| CCN1 | 3491 | 2.898 | 3.099 | Up |
| CD44 | 960 | 4.669 | 4.96 | Up |
| CD99 | 4267 | 2.926 | 6.473 | Up |
| CDC42EP4 | 23580 | 3.055 | 7.619 | Up |
| CEBPB | 1051 | 4.609 | 6.151 | Up |
| CHPT1 | 56994 | 4.71 | 4.288 | Up |
| CHST3 | 9469 | 2.773 | 5.275 | Up |
| CHSY1 | 22856 | 4.75 | 3.325 | Up |
| CLIC1 | 1192 | 2.692 | 5.988 | Up |
| CREBBP | 1387 | 3.298 | 3.605 | Up |
| CSNK1A1 | 1452 | 2.762 | 3.989 | Up |
| CUL4A | 8451 | 2.784 | 3.324 | Up |
| DAAM1 | 23002 | 3.243 | 4.406 | Up |
| DNAJB6 | 10049 | 4.386 | 3.458 | Up |
| DSE | 29940 | 4.024 | 4.922 | Up |
| DTNA | 1837 | 4.32 | 7.678 | Up |
| EFNA1 | 1942 | 2.671 | 5.603 | Up |
| EIF4EBP1 | 1978 | 2.848 | 2.869 | Up |
| EMP1 | 2012 | 3.599 | 6.949 | Up |
| EMP3 | 2014 | 3.846 | 6.083 | Up |
| EPHX1 | 2052 | 4.16 | 2.875 | Up |
| ERBIN | 55914 | 3.515 | 8.183 | Up |
| EZR | 7430 | 3.424 | 7.276 | Up |
| F3 | 2152 | 4.493 | 4.082 | Up |
| FBXO2 | 26232 | 3.19 | 3.264 | Up |
| FERMT2 | 10979 | 2.833 | 4.552 | Up |
| FGF2 | 2247 | 3.581 | 6.105 | Up |
| FLCN | 201163 | 3.567 | 6.307 | Up |
| FYN | 2534 | 3.152 | 3.636 | Up |
| GATAD2A | 54815 | 3.056 | 2.902 | Up |
| GEM | 2669 | 3.23 | 7.414 | Up |
| GJA1 | 2697 | 2.836 | 8.395 | Up |
| GMNN | 51053 | 3.269 | 2.971 | Up |
| GNAI3 | 2773 | 2.758 | 3.703 | Up |
| HIF1A | 3091 | 3.556 | 4.284 | Up |
| HLA-F | 3134 | 3.004 | 5.328 | Up |
| HMBOX1 | 79618 | 2.813 | 8.753 | Up |
| HSPB8 | 26353 | 2.93 | 8.622 | Up |
| IFITM1 | 8519 | 3.11 | 5.837 | Up |
| IFITM2 | 10581 | 4.596 | 5.2 | Up |
| IFITM3 | 10410 | 2.81 | 5.473 | Up |
| IGFBP7 | 3490 | 4.056 | 4.422 | Up |
| IRF9 | 10379 | 3.384 | 3.293 | Up |
| IRS2 | 8660 | 5.629 | 3.27 | Up |
| ISG20 | 3669 | 3.232 | 3.374 | Up |
| KLF6 | 1316 | 2.736 | 5.787 | Up |
| KLHL21 | 9903 | 3.753 | 5.554 | Up |
| LAMC1 | 3915 | 2.711 | 7.313 | Up |
| LAPTM4A | 9741 | 3.153 | 3.671 | Up |
| MAFB | 9935 | 3.474 | 3.253 | Up |
| MAFF | 23764 | 3.436 | 5.304 | Up |
| MAP3K5 | 4217 | 2.701 | 4.836 | Up |
| MARK3 | 4140 | 3.605 | 2.916 | Up |
| MATN2 | 4147 | 3.568 | 4.14 | Up |
| MCL1 | 4170 | 3.653 | 6 | Up |
| MGA | 23269 | 2.797 | 4.151 | Up |
| MKNK2 | 2872 | 3.804 | 4.932 | Up |
| MTHFD2 | 10797 | 4.246 | 2.983 | Up |
| NFE2L2 | 4780 | 3.417 | 6.33 | Up |
| NFKBIA | 4792 | 2.893 | 9.043 | Up |
| NOTCH2 | 4853 | 3.308 | 7.789 | Up |
| OGFRL1 | 79627 | 2.995 | 6.43 | Up |
| PABPC3 | 5042 | 3.213 | 4.295 | Up |
| PALLD | 23022 | 3.489 | 9.085 | Up |
| PARP4 | 143 | 2.769 | 6.973 | Up |
| PDGFC | 56034 | 3.03 | 3.251 | Up |
| PDLIM1 | 9124 | 3.571 | 4.166 | Up |
| PIM1 | 5292 | 3.834 | 4.423 | Up |
| PITPNC1 | 26207 | 2.669 | 6.123 | Up |
| PLAAT4 | 5920 | 2.982 | 6.248 | Up |
| PLBD1 | 79887 | 2.678 | 3.86 | Up |
| PLIN2 | 123 | 3.346 | 3.958 | Up |
| PLP2 | 5355 | 4.772 | 6.115 | Up |
| PLSCR1 | 5359 | 2.928 | 5.037 | Up |
| PRDX6 | 9588 | 3.711 | 5.909 | Up |
| PSMB8 | 5696 | 4.595 | 4.289 | Up |
| PSMB9 | 5698 | 2.722 | 5.015 | Up |
| PSPH | 5723 | 2.896 | 5.841 | Up |
| PTTG1IP | 754 | 2.795 | 6.164 | Up |
| RAB13 | 5872 | 3.842 | 6.653 | Up |
| RAB31 | 11031 | 3.397 | 4.183 | Up |
| RHOQ | 23433 | 2.95 | 9.61 | Up |
| RIPK2 | 8767 | 2.881 | 2.847 | Up |
| RNF114 | 55905 | 3.495 | 3.189 | Up |
| ROM1 | 6094 | 2.959 | 5.32 | Up |
| S100A10 | 6281 | 3.139 | 4.376 | Up |
| S100A6 | 6277 | 3.375 | 3.912 | Up |
| S1PR1 | 1901 | 3.304 | 4.788 | Up |
| SDC2 | 6383 | 3.536 | 4.678 | Up |
| SEC14L1 | 6397 | 2.86 | 4.496 | Up |
| SGK1 | 6446 | 3.116 | 3.333 | Up |
| SLC1A3 | 6507 | 3.626 | 5.579 | Up |
| SLC38A2 | 54407 | 3.54 | 3.712 | Up |
| SLC39A1 | 27173 | 2.808 | 4.6 | Up |
| SLC5A3 | 6526 | 3.65 | 5.317 | Up |
| SLC7A11 | 23657 | 3.251 | 5.246 | Up |
| SLCO4A1 | 28231 | 2.726 | 4.398 | Up |
| SORBS1 | 10580 | 4.057 | 10.424 | Up |
| SOX9 | 6662 | 2.985 | 6.907 | Up |
| SP110 | 3431 | 2.695 | 7.062 | Up |
| SQSTM1 | 8878 | 3.595 | 2.823 | Up |
| STOM | 2040 | 3.944 | 7.424 | Up |
| SWAP70 | 23075 | 3.508 | 6.447 | Up |
| TAGLN2 | 8407 | 3.68 | 4.852 | Up |
| TGIF1 | 7050 | 2.979 | 4.333 | Up |
| TGM2 | 7052 | 2.677 | 3.451 | Up |
| TIMP1 | 7076 | 3.414 | 3.692 | Up |
| TM4SF1 | 4071 | 2.816 | 4.347 | Up |
| TNFRSF1A | 7132 | 4.131 | 6.592 | Up |
| TNFRSF1B | 7133 | 2.817 | 5.589 | Up |
| TOB2 | 10766 | 2.923 | 7.882 | Up |
| TPD52L1 | 7164 | 2.902 | 6.912 | Up |
| TRAM2 | 9697 | 3.098 | 3.98 | Up |
| TRIM8 | 81603 | 2.986 | 5.382 | Up |
| TUBB6 | 84617 | 3.942 | 3.059 | Up |
| UBR5 | 51366 | 3.149 | 4.484 | Up |
| UNG | 7374 | 2.718 | 6.311 | Up |
| VAT1 | 10493 | 2.789 | 4.593 | Up |
| VIM | 7431 | 3.167 | 5.038 | Up |
| WBP1L | 54838 | 2.947 | 3.536 | Up |
| XBP1 | 7494 | 3.835 | 2.955 | Up |
| YBX3 | 8531 | 3.971 | 5.856 | Up |
| ZFP36 | 7538 | 2.77 | 6.013 | Up |
| ABT1 | 29777 | -2.895 | -3.753 | Down |
| ACTR1B | 10120 | -2.818 | -5.079 | Down |
| AGPAT4 | 56895 | -3.02 | -3.018 | Down |
| ANAPC15 | 25906 | -3.271 | -6.353 | Down |
| APEH | 327 | -2.997 | -5.62 | Down |
| APEX1 | 328 | -3.136 | -2.561 | Down |
| ARF5 | 381 | -3.22 | -6.366 | Down |
| ARFGAP2 | 84364 | -3.758 | -4.462 | Down |
| ASH2L | 9070 | -3.168 | -6.32 | Down |
| ASL | 435 | -3.182 | -4.444 | Down |
| ATP7B | 540 | -2.769 | -2.467 | Down |
| BEX3 | 27018 | -3.332 | -4.055 | Down |
| C11orf1 | 64776 | -3.032 | -6.427 | Down |
| C14orf132 | 56967 | -3.939 | -4.474 | Down |
| CBLN1 | 869 | -2.724 | -2.764 | Down |
| CCDC85B | 11007 | -4.454 | -5.417 | Down |
| CDHR1 | 92211 | -2.903 | -2.648 | Down |
| CEP72 | 55722 | -2.888 | -2.497 | Down |
| CES2 | 8824 | -3.087 | -4.791 | Down |
| CHMP6 | 79643 | -3.072 | -3.854 | Down |
| CLASP2 | 23122 | -2.681 | -4.393 | Down |
| COMMD4 | 54939 | -2.844 | -3.443 | Down |
| COQ6 | 51004 | -2.856 | -4.891 | Down |
| CTNNBIP1 | 56998 | -4.658 | -5.185 | Down |
| DCAF11 | 80344 | -3.05 | -6.644 | Down |
| DCTN1 | 1639 | -2.862 | -5.634 | Down |
| DCTN2 | 10540 | -3.304 | -4.462 | Down |
| DCTPP1 | 79077 | -3.72 | -6.933 | Down |
| DEPDC5 | 9681 | -2.73 | -4.355 | Down |
| DHCR24 | 1718 | -3.163 | -3.956 | Down |
| DNPH1 | 10591 | -2.811 | -4.604 | Down |
| DOP1A | 23033 | -3.924 | -2.766 | Down |
| DYNC1I2 | 1781 | -4.467 | -2.513 | Down |
| EIF3K | 27335 | -2.782 | -3.241 | Down |
| EIPR1 | 7260 | -3.768 | -5.583 | Down |
| EPB41L3 | 23136 | -3.214 | -2.891 | Down |
| FAAH | 2166 | -3.173 | -2.591 | Down |
| FECH | 2235 | -3.256 | -5.568 | Down |
| FRAS1 | 80144 | -3.001 | -4.102 | Down |
| FXR2 | 9513 | -2.969 | -2.492 | Down |
| GAMT | 2593 | -3.299 | -2.729 | Down |
| GLT8D2 | 83468 | -3.187 | -5.37 | Down |
| GNAI1 | 2770 | -2.75 | -2.476 | Down |
| GPATCH2 | 55105 | -4.461 | -5.766 | Down |
| GSS | 2937 | -4.204 | -7.499 | Down |
| GUCA1A | 2978 | -2.95 | -3.599 | Down |
| HMGCR | 3156 | -3.302 | -5.238 | Down |
| HMGCS1 | 3157 | -4.085 | -6.449 | Down |
| HPF1 | 54969 | -3.179 | -2.619 | Down |
| IFT27 | 11020 | -3.284 | -5.795 | Down |
| INPP5J | 27124 | -2.771 | -3.093 | Down |
| IVD | 3712 | -3.569 | -2.929 | Down |
| KIF22 | 3835 | -4.108 | -3.626 | Down |
| KLHL7 | 55975 | -2.865 | -6.096 | Down |
| LANCL1 | 10314 | -4.644 | -4.427 | Down |
| LETMD1 | 25875 | -3.091 | -2.433 | Down |
| LRRC49 | 54839 | -2.683 | -3.496 | Down |
| MAGEH1 | 28986 | -3.146 | -5.243 | Down |
| MECR | 51102 | -2.869 | -6.628 | Down |
| MFN2 | 9927 | -2.708 | -4.42 | Down |
| MIS18A | 54069 | -3.222 | -3.851 | Down |
| MPP2 | 4355 | -3.219 | -5.824 | Down |
| MRPL4 | 51073 | -2.799 | -5.456 | Down |
| MYL5 | 4636 | -3.132 | -2.919 | Down |
| NABP2 | 79035 | -3.278 | -3.904 | Down |
| NDUFB2 | 4708 | -3.127 | -3.854 | Down |
| NDUFB8 | 4714 | -3.492 | -2.792 | Down |
| NIPSNAP1 | 8508 | -3.024 | -6.432 | Down |
| NLGN3 | 54413 | -4.127 | -3.098 | Down |
| NUDT2 | 318 | -3.721 | -7.375 | Down |
| PARP2 | 10038 | -2.889 | -3.91 | Down |
| PCYT2 | 5833 | -3.48 | -2.664 | Down |
| PDE2A | 5138 | -3.069 | -4.434 | Down |
| PEX1 | 5189 | -3.24 | -2.688 | Down |
| PEX7 | 5191 | -3.245 | -3.978 | Down |
| PGRMC1 | 10857 | -3.794 | -7.202 | Down |
| PNKP | 11284 | -3.031 | -4.187 | Down |
| POLB | 5423 | -3.026 | -6.402 | Down |
| POLR2A | 5430 | -2.867 | -4.805 | Down |
| POLR2G | 5436 | -3.65 | -4.201 | Down |
| POMGNT1 | 55624 | -3.444 | -2.879 | Down |
| PPOX | 5498 | -3.832 | -2.487 | Down |
| PPP2R1A | 5518 | -2.711 | -5.047 | Down |
| PRKAG1 | 5571 | -3.29 | -5.841 | Down |
| PTPRE | 5791 | -3.335 | -3.982 | Down |
| RAB33A | 9363 | -2.739 | -4.166 | Down |
| RABGGTA | 5875 | -3.196 | -3.199 | Down |
| RBM3 | 5935 | -2.773 | -3.627 | Down |
| RBX1 | 9978 | -3.141 | -2.8 | Down |
| ROGDI | 79641 | -5.025 | -6.104 | Down |
| RTCA | 8634 | -3.027 | -5.256 | Down |
| RTN3 | 10313 | -2.696 | -4.506 | Down |
| SC5D | 6309 | -4.14 | -4.44 | Down |
| SEC61A2 | 55176 | -3.315 | -7.005 | Down |
| SIRT5 | 23408 | -3.529 | -4.479 | Down |
| SLC25A11 | 8402 | -3.436 | -7.044 | Down |
| SLC25A15 | 10166 | -3.45 | -5.827 | Down |
| SMIM8 | 57150 | -3.004 | -5.213 | Down |
| SMUG1 | 23583 | -2.793 | -8.779 | Down |
| SNX4 | 8723 | -3.115 | -4.58 | Down |
| SPAG7 | 9552 | -3.03 | -4.086 | Down |
| SQLE | 6713 | -4.499 | -4.349 | Down |
| ST3GAL5 | 8869 | -3.07 | -3.104 | Down |
| STK16 | 8576 | -3.884 | -3.041 | Down |
| STMN1 | 3925 | -3.037 | -4.189 | Down |
| STX12 | 23673 | -3.273 | -4.501 | Down |
| TARBP1 | 6894 | -5.852 | -3.346 | Down |
| TEDC2 | 80178 | -2.662 | -2.959 | Down |
| TM7SF2 | 7108 | -3.028 | -7.456 | Down |
| TMEM62 | 80021 | -2.905 | -3.128 | Down |
| TRAPPC2L | 51693 | -4.609 | -7.416 | Down |
| TRMT61B | 55006 | -3.294 | -3.783 | Down |
| TRPC1 | 7220 | -2.698 | -3.919 | Down |
| USP5 | 8078 | -3.622 | -5.02 | Down |
| UVRAG | 7405 | -2.833 | -2.552 | Down |
| VBP1 | 7411 | -3.148 | -2.908 | Down |
| VPS39 | 23339 | -2.724 | -2.985 | Down |
| WASF1 | 8936 | -2.83 | -4.036 | Down |
| WDR13 | 64743 | -2.928 | -3.341 | Down |
| WDR61 | 80349 | -2.87 | -2.425 | Down |
| ZC4H2 | 55906 | -3.65 | -4.693 | Down |
| ZNF174 | 7727 | -2.961 | -4.671 | Down |

# Supplementary Table 2. ALD-related genes that can be searched in GeneCard.

| Symbol | Entrez ID | Z-score | | Relevance Score |
| --- | --- | --- | --- | --- |
|  |  | ALD | AD |  |
| ABCD1 | 215 | -0.882 | -0.134 | 68.992 |
| ABCD2 | 225 | NA | NA | 20.13 |
| ABCD3 | 5825 | -1.232 | 1.128 | 19.498 |
| ABCD4 | 5826 | NA | NA | 15.724 |
| EHHADH | 1962 | -0.182 | -0.14 | 7.461 |
| PEX16 | 9409 | -2.064 | -5.024 | 7.161 |
| SLC25A17 | 10478 | -2.108 | -4.309 | 7.103 |
| HSD17B4 | 3295 | NA | NA | 6.982 |
| PEX19 | 5824 | -2.56 | -6.207 | 6.982 |
| PEX2 | 5828 | -1.548 | 2.724 | 6.922 |
| PEX1 | 5189 | -3.24 | -2.688 | 6.776 |
| PEX10 | 5192 | -3.141 | -1.823 | 6.776 |
| PEX12 | 5193 | NA | NA | 6.776 |
| PEX13 | 5194 | -1.714 | -2.335 | 6.776 |
| PEX26 | 55670 | NA | NA | 6.776 |
| PEX5 | 5830 | -0.963 | -2.375 | 6.776 |
| PEX6 | 5190 | 0.763 | -1.097 | 6.776 |
| SLC27A2 | 11001 | -1.952 | -5.451 | 5.263 |
| ELOVL1 | 64834 | NA | NA | 4.747 |
| ABCB6 | 10058 | NA | NA | 4.711 |
| BCAP31 | 10134 | 1.946 | -1.954 | 4.592 |
| MOG | 4340 | NA | NA | 4.44 |
| ACOX1 | 51 | 1.847 | -0.923 | 4.439 |
| SCP2 | 6342 | NA | NA | 4.342 |
| PPARA | 5465 | NA | NA | 4.32 |
| PEX7 | 5191 | NA | NA | 4.029 |
| HMGB1 | 3146 | -1.113 | 0.93 | 3.784 |
| GATAD1 | 57798 | -1.849 | 3.318 | 2.747 |
| PEX11B | 8799 | -2.382 | -4.313 | 2.747 |
| PEX14 | 5195 | 0.102 | -3.037 | 2.747 |
| PEX3 | 8504 | NA | NA | 2.747 |
| PLCH2 | 9651 | NA | NA | 2.747 |
| SOAT1 | 6646 | NA | NA | 2.198 |

# Supplementary Table 3. PPI network analysis conducted using STRING and MCODE

| Symbol | Entrez ID | Node Cluster | MCODE Score | Leading Edge Gene | Degree |
| --- | --- | --- | --- | --- | --- |
| APOE | 348 | Seed | 6 | FALSE | 8 |
| ANXA5 | **308** | **Clustered** | **6** | **TRUE** | **9** |
| B2M | **567** | **Clustered** | **5.79** | **TRUE** | **10** |
| CD44 | **960** | **Clustered** | **5.79** | **TRUE** | **30** |
| FGF2 | **2247** | **Clustered** | **5.79** | **TRUE** | **21** |
| CCL2 | 6347 | Clustered | 5.79 | FALSE | 9 |
| SDC1 | 6382 | Clustered | 5.79 | FALSE | 19 |
| STAT3 | 6774 | Clustered | 5.79 | FALSE | 41 |
| SDC4 | 6385 | Clustered | 5 | FALSE | 17 |
| SDC2 | 6383 | Unclustered | 4.76 | TRUE | 20 |
| ABCA1 | 19 | Unclustered | 3 | TRUE | 7 |
| CASP7 | 840 | Unclustered | 3 | TRUE | 7 |
| GJA1 | 2697 | Unclustered | 3 | TRUE | 9 |
| IRF9 | 10379 | Unclustered | 3 | TRUE | 8 |
| S1PR1 | 1901 | Unclustered | 3 | TRUE | 8 |
| FYN | 2534 | Unclustered | 2.7 | TRUE | 31 |
| HLA-F | 3134 | Unclustered | 2.7 | TRUE | 5 |
| NFE2L2 | 4780 | Unclustered | 2.7 | TRUE | 9 |
| PSMB8 | 5696 | Unclustered | 2.7 | TRUE | 11 |
| PSMB9 | 5698 | Unclustered | 2.7 | TRUE | 9 |
| NOTCH2 | 4853 | Unclustered | 2.4 | TRUE | 4 |
| ALDH2 | 217 | Unclustered | 2 | TRUE | 6 |
| GMNN | 51053 | Unclustered | 2 | TRUE | 19 |
| FOXM1 | 2305 | Unclustered | 2 | FALSE | 49 |
| FZD7 | 8324 | Unclustered | 2 | FALSE | 5 |
| UBE2L6 | 9246 | Unclustered | 2 | FALSE | 3 |
| EZR | 7430 | Unclustered | 1.86 | TRUE | 15 |
| XPO1 | 7514 | Unclustered | 1.67 | FALSE | 18 |
| YAP1 | 10413 | Unclustered | 1.04 | FALSE | 9 |
| DAAM1 | 23002 | Unclustered | 0.67 | TRUE | 5 |
| FERMT2 | 10979 | Unclustered | 0.67 | TRUE | 4 |
| ENAH | 55740 | Unclustered | 0.67 | FALSE | 5 |
| G3BP1 | 10146 | Unclustered | 0.67 | FALSE | 5 |
| GLUL | 2752 | Unclustered | 0.67 | FALSE | 4 |
| GNA12 | 2768 | Unclustered | 0.67 | FALSE | 7 |
| PABPC1 | 26986 | Unclustered | 0.67 | FALSE | 17 |
| TNPO1 | 3842 | Unclustered | 0.67 | FALSE | 6 |
| DSE | 29940 | Unclustered | 0 | TRUE | 3 |
| PRDX6 | 9588 | Unclustered | 0 | TRUE | 4 |
| GNG5 | 2787 | Unclustered | 0 | FALSE | 6 |

**Supplementary Table 4. RNA-seq dataset of AD**

| **ArrayExpress ID** | **Source** | **Platform** | **AD** | **Control** | **Total** |
| --- | --- | --- | --- | --- | --- |
| [Series GSE53699](https://www.ncbi.nlm.nih.gov/geo/query/acc.cgi?acc=GSE53699) | Postmortem  brain | Illumina HiSeq 2500 | 9 | 8 | 17 |
| [Series GSE53697](https://www.ebi.ac.uk/arrayexpress/experiments/E-GEOD-53697/?keywords=%22alzheimer%27s%20disease%22%20AND%20%22brain%22&organism=Homo%20sapiens&exptype%5B0%5D=&exptype%5B1%5D=&sortby=type&sortorder=ascending) | Postmortem  brain | Illumina HiSeq 2500 | 18 | 16 | 34 |
| [Series GSE67333](https://www.ncbi.nlm.nih.gov/geo/query/acc.cgi?acc=GSE67333) | Postmortem  brain | Illumina HiSeq 2000 | 4 | 4 | 8 |
| [Series GSE184942](https://www.ncbi.nlm.nih.gov/geo/query/acc.cgi?acc=GSE184942) | Postmortem  brain | Illumina HiSeq 2000 | 5 | 5 | 10 |
| [Series GSE159699](https://www.ncbi.nlm.nih.gov/geo/query/acc.cgi?acc=GSE159699) | Postmortem  brain | Illumina NextSeq 500 | 12 | 18 | 30 |
| Series GSE153875 | Postmortem  brain | Illumina NextSeq 500 | 11 | 18 | 29 |
| [Series GSE104704](https://www.ncbi.nlm.nih.gov/geo/query/acc.cgi?acc=GSE104704) | Postmortem  brain | Illumina NextSeq 500 | 12 | 18 | 30 |
|  |  |  | **71** | **87** | **158** |
